# Immunohistochemical Evaluation of BARX1, DLX4, FOXE1, HOXB3, and MSX2 in Nonsyndromic Cleft Affected Tissue

**DOI:** 10.15388/Amed.2022.29.2.13

**Published:** 2022-06-29

**Authors:** Mārtiņš Vaivads, Ilze Akota, Māra Pilmane

**Affiliations:** Institute of Anatomy and Anthropology, Riga Stradins University, Riga, Latvia; Department of Oral and Maxillofacial Surgery, Riga Stradins University, Riga, Latvia; Cleft Lip and Palate Centre, Institute of Stomatology, Riga Stradins University, Riga, Latvia; Institute of Anatomy and Anthropology, Riga Stradins University, Riga, Latvia

**Keywords:** cleft lip, cleft palate, homeobox genes, cleft candidate genes

## Abstract

**Background:**

Nonsyndromic craniofacial clefts are relatively common congenital malformations which could create a significant negative effect on the health status and life quality of affected individuals within the pediatric population. Multiple cleft candidate genes and their coded proteins have been described with their possible involvement during cleft formation. Some of these proteins like Homeobox Protein BarH-like 1 (BARX1), Distal-Less Homeobox 4 (DLX4), Forkhead Box E1 (FOXE1), Homeobox Protein Hox-B3 (HOXB3), and Muscle Segment Homeobox 2 (MSX2) have been associated with the formation of craniofacial clefts. Understanding the pathogenetic mechanisms of nonsyndromic craniofacial cleft formation could provide a better knowledge in cleft management and could be a possible basis for development and improvement of cleft treatment options. This study investigates the presence of BARX1, DLX4, FOXE1, HOXB3, and MSX2 positive cells by using immunohistochemistry in different types of cleft-affected tissue while determining their possible connection with cleft pathogenesis process.

**Materials and Methods:**

Craniofacial cleft tissue material was obtained during cleft-correcting surgery from patients with nonsyndromic craniofacial cleft diagnosis. Tissue material was gathered from patients who had unilateral cleft lip (n=36), bilateral cleft lip (n=13), and cleft palate (n=26). Control group (n=7) tissue material was received from individuals without any craniofacial clefts. The number of factor positive cells in the control group and patient group tissue was evaluated by using the semiquantitative counting method. Data was evaluated with the use of nonparametric statistical methods.

**Results:**

Statistically significant differences were identified between the number of BARX1, FOXE1, HOXB3, and MSX2-containing cells in controls and cleft patient groups but no statistically significant difference was found for DLX4. Statistically significant correlations between the evaluated factors were also notified in cleft patient groups.

**Conclusions:**

HOXB3 could be more associated with morphopathogenesis of unilateral cleft lip during postnatal course of the disorder. FOXE1 and BARX1 could be involved with both unilateral and bilateral cleft lip morphopathogenesis. The persistence of MSX2 in all evaluated cleft types could indicate its possible interaction within multiple cleft types. DLX4 most likely is not involved with postnatal cleft morphopathogenesis process.

## Santrauka.

**Kontekstas:**

Nesindrominis veido ir kaukolės nesuaugimas yra santykinai dažna įgimta anomalija, galinti turėti reikšmingų neigiamų pasekmių pediatrinės populiacijos asmenų sveikatos būklei ir gyvenimo kokybei. Aprašyta daugelis genų, kurie potencialiai gali lemti nesuaugimus, ir jų koduojami baltymai, aptariamas galimas jų dalyvavimas susidarant nesuaugimui. Dalis šių baltymų – tokie kaip homeozinis baltymas BarH-tipas 1 (BARX1), distalinis homeozinis genas 4 (DLX4), FOX E1 genas (FOXE1), homeozinis baltymas Hox-B3 (HOXB3) bei raumenų segmento homeozinis 2 genas (MSX2) – buvo siejami su kaukolės ir veido nesuaugimais. Patogeninio nesindrominio kaukolės ir veido nesuaugimo suvokimas galėtų suteikti daugiau žinių, kaip vertinti su nesuaugimais susijusius atvejus bei galimai taptų pradžios tašku, iš kurio kiltų naujos ar tobulesnės nesuaugimo gydymo alternatyvos. Šiame tyrime nagrinėjama, ar yra BARX1, DLX4, FOXE1, HOXB3 ir MSX2 genų turinčių ląstelių, pasitelkiant imunohistochemiją ir nagrinėjant įvairius nesuaugimo paveiktų audinių tipus, siekiant nustatyti galimą jų ryšį su nesuaugimo patogeneze.

**Medžiagos ir metodai:**

Kaukolės ir veido nesuaugimo audinio medžiaga buvo įgyta nesuaugimo korekcinės operacijos metu iš pacientų, kuriems buvo nustatyta nesindrominio kaukolės ir veido nesuaugimo diagnozė. Audinio medžiaga buvo surinkta iš pacientų, kuriems yra vienpusis (n = 36) bei dvipusis lūpos nesuaugimas (n = 13) bei nesuaugęs gomurys (n = 26). Kontrolinės grupės (n = 7) audinio medžiaga gauta iš asmenų, neturinčių jokių kaukolės ar veido nesuaugimų. Teigiamo tyrimo faktoriaus ląstelių skaičius kontrolinės grupės ir pacientų grupės audiniuose buvo vertinamas pusiau kiekybiniu skaičiavimo metodu. Duomenys vertinti pasitelkus neparametrinius statistinius metodus.

**Rezultatai:**

Buvo nustatyta statistiškai reikšmingų skirtumų tarp ląstelių, turinčių BARX1, FOXE1, HOXB3 bei MSX2, skaičiaus kontrolinėje grupėje bei pacientų, kurių audinys nesuaugęs, grupėse, tačiau nebuvo nustatyta jokių statistiškai reikšmingų skirtumų dėl DLX4. Statistiškai reikšmingos vertinamų faktorių koreliacijos buvo nustatytos ir pacientų, kurių audiniai nesuaugę, grupėse.

**Išvados:**

HOXB3 galėtų būti labiau siejamas su vienpusio lūpos nesuaugimo morfopatogeneze po gimimo. FOXE1 ir BARX1 galima raiška tiek vienpusio, tiek ir abipusio lūpos nesuaugimo morfopatogenezėje. Kadangi MSX2 sistemingai yra visų nagrinėjamų nesuaugimo tipų atveju, galima daryti išvadą, kad MSX2 galimai yra susijęs su daugeliu nesuaugimo tipų. DLX4 veikiausiai nėra susijęs su nesuaugimo morfopatogenezės po gimimo procesu.

## Introduction

Craniofacial clefts are characterized as abnormalities of the facial region development in which the process of facial prominence fusion has been defective and incomplete during the embryogenesis [1]. Craniofacial clefts when compared to other inborn anomalies have been described as relatively common congenital pathologies with the incidence of 1 per 700 live births within the global population [2].

The formation of craniofacial clefts can be affected by multiple factors. Factors from the external environment like physical factors, chemical factors, biological factors have been previously described as contributing agents which could increase the likelihood of incorrect development of the facial region [3]. Genetic factors have also been described as a part of the pathogenetic mechanisms of orofacial cleft formation. Genes possibly involved with pathogenesis of orofacial clefts are called cleft candidate genes. Multiple cleft candidate genes have been previously described as directly associated or involved with the formation of syndromic and nonsyndromic orofacial cleft cases [4].

Orofacial clefts can be classified by their location. Clefts which affect the lip can be classified as unilateral or bilateral cleft lip. Clefts which affect the palatal region can be described as isolated cleft palate or a combination of cleft palate with cleft lip. Multiple classification systems have been used to classify orofacial clefts [5, 6]. The tissue in lip and the palate differs during the developmental process because of the involvement of different regulatory genes and transcription factors within each of these specific craniofacial regions. Due to the differences of specific gene and transcription factor activation and regulation in different orofacial regions, the oral cavity epithelium and the underlying connective tissue undergo multiple changes and distinctions during the development within these craniofacial regions of the upper lip and the palate [1, 7, 8].

Homeobox Protein BarH-like 1 (BARX1) is a transcription factor involved with the development and positioning of mesenchyme in multiple organs like the stomach, the spleen and the branchial arch region while also affecting the differentiation of the endodermal surface epithelium in stomach and tooth development process [9, 10]. BARX1 is involved with the regulatory development mechanisms in the craniofacial region like the developing palatal region [10]. BARX1 has been previously associated with the formation of craniofacial clefts in humans [11].

Distal-Less Homeobox 4 (DLX4) protein is a transcription factor involved with the differentiation of cranial neural crest cells, cranial mesenchyme and together with other DLX transcription factors are involved with the development and patterning of the craniofacial region tissue and organs like the teeth and the developing jaw region [12, 13]. DLX4 involvement has been described in pathologies with improper regulation of cell proliferation and differentiation like breast cancer, ovarian cancer and leukemia [14]. DLX4 has been previously associated with the formation of orofacial clefts [15].

Forkhead Box E1 (FOXE1) protein is a transcription factor involved with the development of thyroid gland and the craniofacial epithelium formation [16]. FOXE1 involvement has been described in pathologies like congenital hypothyroidism, Bamforth syndrome and cleft lip and palate [17]. FOXE1 has been associated with craniofacial cleft development in humans [18].

Homeobox Protein Hox-B3 (HOXB3) has been described as a factor involved with the development of craniofacial region epithelium like the pharyngeal epithelium and also regulates neural crest cell migration in the craniofacial region [19, 20]. HOXB3 gene also regulates capillary tube formation within the developing embryonic connective tissue together with other HOX genes [21]. HOXB3 involvement has been discussed in multiple pathologies with disrupted tissue growth and proliferation including clefts, disrupted hemopoiesis and cancer formation [19, 22].

Muscle Segment Homeobox 2 (MSX2) has been described as regulatory factor for pluripotent stem cell differentiation into mesodermal tissue, regulates epithelial and mesenchymal tissue interactions and the development of the limbs and the craniofacial region [23]. The role of MSX2 has been notified in case of some craniofacial pathologies, like craniosynostosis and abnormalities with the formation of parietal and frontal bones [23, 24]. MSX2 has been associated with the formation of orofacial clefts [25].

BARX1, DLX4, FOXE1, HOXB3, and MSX2 are transcription factors which regulate the development of the orofacial region and patterning process within craniofacial tissue. BARX1, DLX4, HOXB3 and MSX2 proteins contain a homeodomain [9, 13, 22, 23], while FOXE1 contains the fork head domain [16] which can bind to deoxyribonucleic acid (DNA) and regulate patterning and regional specification in craniofacial tissue. Genes encoding these transcription factors have been previously associated with the formation of craniofacial clefts. These specific factors have been chosen for our study because they have specific DNA-binding homeodomains or fork head domains, and regulate different aspects of craniofacial tissue development..

The aim of this study is to determine the presence of BARX1, DLX4, FOXE1, HOXB3, and MSX2 proteins in the epithelium and the underlying connective tissue within the postnatal cleft affected tissue groups in comparison to controls and to evaluate the correlations between the factors within the nonsyndromic cleft affected tissue. This specific combination of cleft candidate gene proteins and their possible interactions has not been described in previous studies within human cleft affected tissue. These specific proteins have been chosen for this study because of their previously described role in formation of orofacial cleft as cleft candidate gene coded proteins and to better understand their presence in cleft affected tissue and to determine their possible representation differences and similarities between the tissues of different cleft types.

## Materials and methods

### Research subjects

All patient and control group tissue samples which were analyzed in this study were donated with a voluntary agreement from the parents of the patients and the parents of the controls. Both the control group and the patient group tissues were acquired in Cleft Lip and Palate Centre of the Institute of Stomatology of Riga Stradins University (RSU). The analysis and study of said tissue material was performed in the RSU Department of Morphology. The approval of the study protocol was received from the RSU Ethics Committee (22.05.2003 – for the acquisition and evaluation of cleft affected tissue material; Nr.6-1/10/11, 24.09.2020 – for the evaluation of specific cleft candidate genes and their coded proteins in cleft affected tissue and control group tissue). This study was conducted in accordance with the Declaration of Helsinki of 1964.

The evaluated patient study groups were split based on the type and location of the cleft affected tissue. Unilateral cleft lip tissue group, bilateral cleft lip tissue group and cleft palate tissue group were formed. The tissue material obtained from the cleft correcting surgery from cleft affected tissue group patients contained oral cavity surface epithelium and the underlying connective tissue.

The inclusion criteria for each patient group were the diagnosis of cleft lip (unilateral or bilateral) or cleft palate, surgery performed to repair said orofacial cleft, no presence of any other orofacial pathology during the time of surgery, the age of the patients during the surgery restricted to a time frame before the moment of primary dentition (from age of 3 months to age of 18 months).

Based on the available tissue material the patient groups were split into 36 patients with unilateral cleft lip (20 male and 16 female patients with an age from 3 months to 8 months), 13 patients with bilateral cleft lip (10 male and 3 female patients with an age from 4 months to 16 months) and 26 patients with cleft palate (18 male and 8 female patients with an age from 4 months to 14 months).

The tissues of the control group were taken from 7 patients who received surgery (labial frenectomy) for the correction of enlarged upper lip frenulum. The control group contained 4 male and 3 female patients with an age from 8 years to 11 years. The inclusion criteria for the control group were the diagnosis of upper lip frenulum hypertrophy, no clefts found during clinical investigation (visual inspection, radiological investigation), no orofacial clefts found in the patient anamnesis or in family anamnesis, no other pathological process like inflammation, fibrosis, malignancy, atrophy present within the collected tissue sample.

Due to the very limited amount of control tissue material available for immunohistochemical evaluation BARX1, FOXE1, HOXB3, MSX2 immunoreactivity could be analyzed only from 5 control tissue group patients while DLX4 immunoreactivity could be evaluated from the tissue material of 7 patients within the control group.

### Immunohistochemistry

The main technique used to prepare and assess the tissue samples for this study was the standard streptavidin and biotin immunohistochemical method, which was utilized to provide the detection of BARX1, DLX4, FOXE1, HOXB3, MSX2 proteins [26]. The control and patient tissues were fixated in 2% formaldehyde together with 0.2% picric acid within a buffer solution containing 0.1 M phosphate. The washing procedure was executed with a phosphate-buffered saline solution which was accommodated with 10% saccharose for at least 12 hours. The embedding procedure was performed in paraffin. Afterwards the paraffinized tissue blocks were cut into multiple thin sections of 6–7 μm each. The procedure of deparaffinization was conducted and the staining of slides was implemented by using biotin-streptavidin immunohistochemical method to ensure the detection cleft candidate gene proteins with antibodies for BARX1 (LS-C29810, 1:100, LifeSpan BioSciences, Inc., Seattle, WA, US), DLX4 (orb160775, 1:100, Biorbyt Ltd., Cambridge, UK), FOXE1 (ab5080, 1:500, Abcam, Cambridge, UK), HOXB3 (sc28606, 1:100, Santa Cruz Biotechnology, Dallas, TX, USA), MSX2 (ab22606, 1:100, Abcam, Cambridge, UK)

Leica DC 300F digital camera was used to provide the visualization of slides. Image Pro Plus software (Media Cybernetics, Inc., Rockville, MD, USA) allowed to process and analyze the collected images.

The relative frequency of BARX1, DLX4, FOXE1, HOXB3, and MSX2 containing cells within the patient and control group slides stained with the biotin-streptavidin immunohistochemical method was obtained by using nonparametric slide evaluation with the semiquantitative counting method [27, 28, 29]. The relative frequency of positively stained structures within the patient group and control group tissue was conducted with light microscopy by evaluating 5 different visual fields within each tissue section by 2 independent and separate researchers. The summary of the designations used for the slide evaluation with the semiquantitative counting method is available in Table 1.

**Table 1.** The designations of relative frequency for immunohistochemically analyzed cleft candidate gene proteins within a visual field.

Designation	Description
0	No factor positive cells
0/+	Rare occurrence of factor positive cells
+	A few factor positive cells
+/++	Few to moderate number of factor positive cells
++	Moderate number of factor positive cells
++/+++	Moderate to numerous factor positive cells
+++	Numerous factor positive cells
+++/++++	Numerous to abundant factor positive cells
++++	Abundance of factor positive cells

### Statistical methods

Descriptive and analytical statistical methods were employed for analysis of data. The count of immunopositive cells within each visual field and descriptive statistics (median value, interquartile range) calculations were utilized. Spearman’s rank correlation coefficient calculation was applied to the data. The strength of correlation for Spearman’s rho (r_s_) value was understood with the following: very weak correlation (r_s_=0.0–0.2), weak correlation (r_s_=0.2–0.4), moderate correlation (r_s_=0.4–0.6), strong correlation (r_s_=0.6–0.8), very strong correlation (r_s_=0.8–1.0). The semiquantitative count of immunopositive cells within the patient group and control group tissues is conveyed as median values. The calculation of statistical significance between the number of immunopositive structures in each research group was accomplished with the Mann–Whitney U test and Kruskal–Wallis H test. SPSS Statistics (version 25.0, IBM Company, Chicago, IL, USA) program was utilized for data statistical analysis. Statistical significance of all statistical calculations was accepted with a p-value of <0.05.

## Results

### BARX1 immunohistochemistry

**Figure 1.**
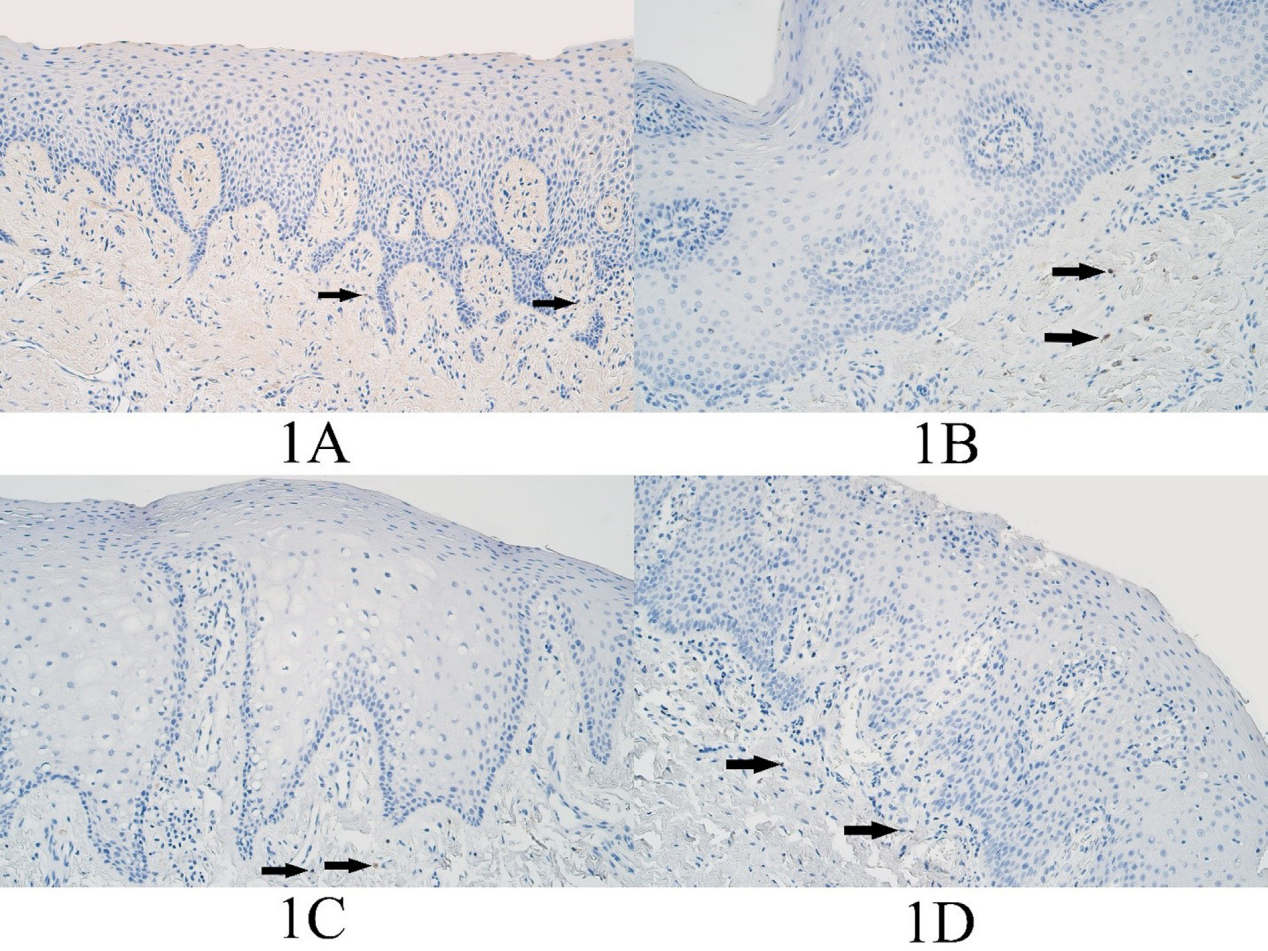
BARX1 immunopositive cells within the control group and cleft affected tissues. (1A) Control tissue lacks BARX1-containing epitheliocytes and shows only a rare occurrence of BARX1 immunopositive connective tissue cells, BARX1 IMH (arrows), 200x. (1B) Unilateral cleft lip patient with an absence of BARX1 containing epitheliocytes and showing a few BARX1 immunopositive connective tissue cells (arrows), BARX1 IMH, 200x. (1C) Bilateral cleft lip patient absent of BARX1-containing epitheliocytes and demonstrating only a few BARX1 immunopositive connective tissue cells (arrows), BARX1 IMH, 200x. (1D) Cleft palate patient absent of BARX1-containing epitheliocytes and showing a rare occurrence of BARX1 positive connective tissue cells (arrows), BARX1 immunohistochemistry (IMH), 200x.

In the control group, the median number of BARX1 positive cells in the epithelium was no immunopositive structures (0) and it ranged from no BARX1 positive cells (0) to 0 positive cells. In the control group connective tissues the median number of BARX1 positive cells was no immunopositive structures (0) and it ranged from no BARX1 positive cells (0) to barely detectable (0/+) positive cells (Figure 1A).

In the unilateral cleft lip group epithelium, the median number of BARX1 positive epitheliocytes was no positive cells (0) and no BARX1 positive epitheliocytes were found within any slide of the unilateral cleft lip tissue group. The median number of BARX1 positive connective tissue cells was a few (+) positive cells and it ranged from no positive cells (0) to few to moderate (+/++) number of BARX1 immunopositive connective tissue cells, mainly macrophages (Figure 1B).

The median number of BARX1 positive epitheliocytes in the bilateral cleft lip tissue group was no positive cells (0) and no BARX1 positive epitheliocytes were found in any slide of the bilateral cleft lip affected tissue group. The median number of BARX1 immunopositive structures in the connective tissues of the bilateral cleft lip group was few to moderate (+/++) and it ranged from no BARX1 positive structures (0) to few to moderate (+/++) positive structures (Figure 1C).

In the cleft palate affected tissue group, the median number of BARX1 positive epitheliocytes was no positive cells (0) and no BARX1 positive epitheliocytes were found in any slide of the cleft palate affected tissue group. In the connective tissues, the median number of BARX1 positive structures was barely detectable to a few (0/+ - +) and ranged from no BARX1 positive cells (0) to few to moderate (+/++) positive structures (Figure 1D).

The Kruskal–Wallis H test revealed that no statistically significant difference was present in the number of BARX1 immunopositive surface epitheliocytes among all four groups (H=0.000, df=3, p=1.000). This test indicated a statistically significant difference in the number of BARX1 positive structures in the connective tissue among all four tissue groups (H=8.511, df=3, p=0.037).

The Mann–Whitney U test indicated no statistically significant difference in the number of BARX1 immunopositive surface epitheliocytes between the controls and the unilateral cleft lip tissue group (U=90.0, p=1.000). This test revealed a statistically significant difference in the number of BARX1 immunopositive connective tissue cells between the controls and the unilateral cleft lip tissue group (U=12.0, p=0.001).

The Mann–Whitney U test indicated no statistically significant difference in the number of BARX1 immunopositive surface epitheliocytes between the controls and the bilateral cleft lip tissue group (U=30.0, p=1.000). This test revealed a statistically significant difference in the number of BARX1 immunopositive connective tissue cells between the controls and the bilateral cleft lip tissue group (U=12.0, p=0.046).

The Mann–Whitney U test revealed no statistically significant difference in the number of BARX1 immunopositive surface epitheliocytes between the controls and the cleft palate tissue group (U=60.0, p=1.000). This test indicated no statistically significant difference in the number of BARX1 immunopositive connective tissue cells between the controls and the cleft palate tissue group (U=39.0, p=0.110).

### DLX4 immunohistochemistry

**Figure 2.**
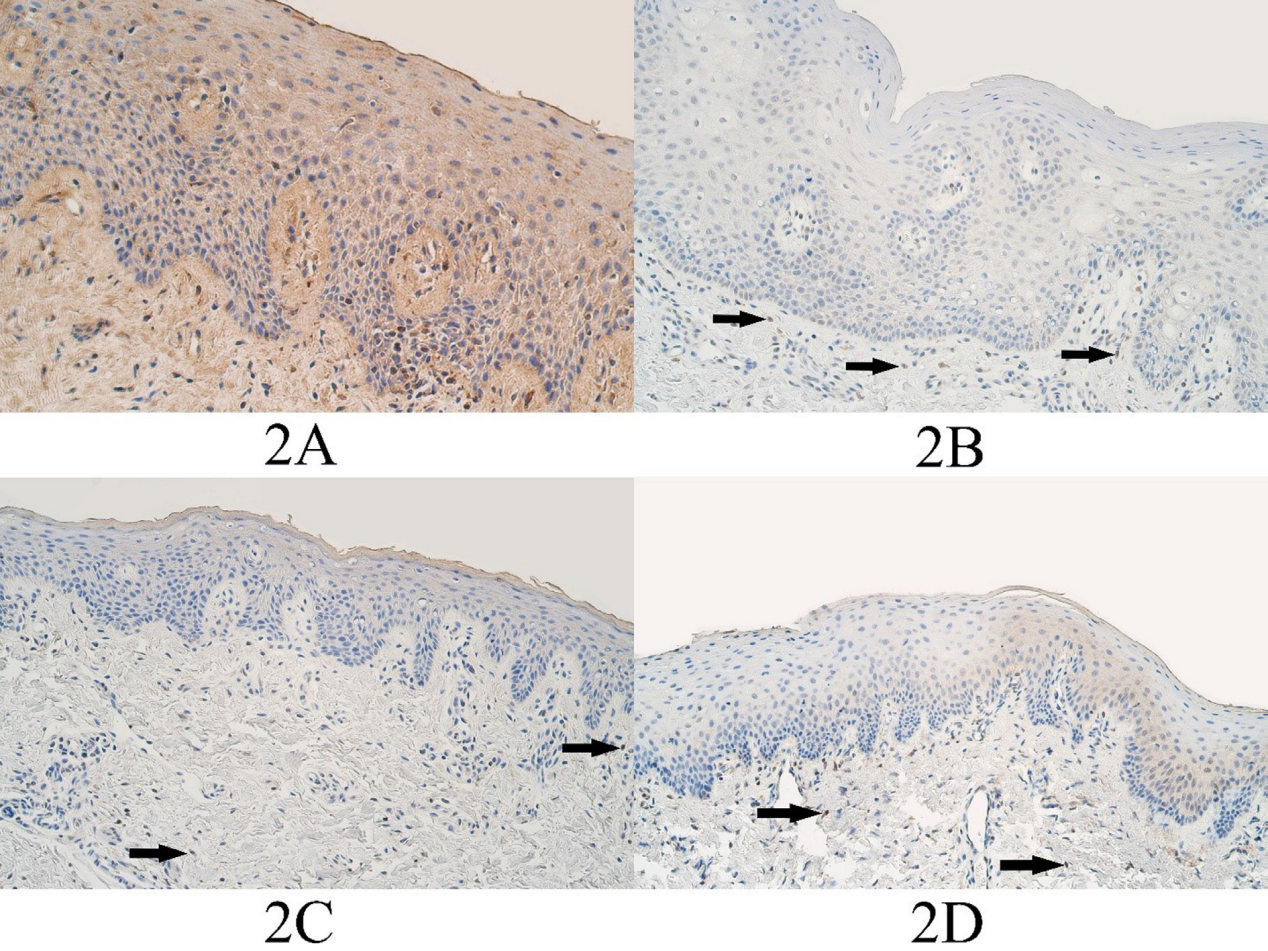
DLX4 immunopositive cells within the control group and cleft affected tissues. (2A) Control group with moderate to numerous DLX4-containing epitheliocytes and connective tissue cells, DLX4 IMH, 200x. (2B) Unilateral cleft lip patient with a few DLX4-containing epitheliocytes and connective tissue cells (arrows), DLX4 IMH, 200x. (2C) Bilateral cleft lip patient with few to moderate DLX4-containing epitheliocytes and connective tissue cells (arrows), DLX4 IMH, 200x. (2D) Cleft palate patient with a moderate number of weakly stained DLX4-containing epitheliocytes and a few DLX4 positive connective tissue cells (arrows), DLX4 IMH, 200x.

The median number of DLX4 positive epitheliocytes in the control group epithelium was a few (+) and it ranged from no positive cells (0) to moderate to numerous (++/+++). The median number of DLX4 positive connective tissue cells was a few (+) and it ranged from barely detectable (0/+) to moderate to numerous (++/+++) (Figure 2A).

In the unilateral cleft lip tissue group the median number of DLX4 positive epitheliocytes was few to moderate (+/++) and it varied from no positive structures (0) to numerous (+++) in the epithelium. The median number of DLX immunopositive structures in the unilateral cleft lip connective tissues was a few (+) positive structures and it varied from no positive structures (0) to moderate to numerous (++/+++) immunopositive structures (Figure 2B).

The median number of DLX4 positive structures in the epithelium of the bilateral cleft lip tissue group was few to moderate (+/++) and it ranged from no positive structures (0) to moderate to numerous (++/+++) immunopositive structures. The median number of DLX4 positive connective tissue cells of the bilateral cleft lip group was few to moderate (+/++) and it ranged from a few (+) positive structures to few to moderate (+/++) immunopositive structures (Figure 2C).

In the cleft palate tissue group the median number of DLX4 positive epitheliocytes was few to moderate – moderate (+/++ - ++) and it ranged from no positive cells (0) to moderate (++) number of DLX4 immunopositive cells. The median number of DLX4 positive cells in the connective tissues of the cleft palate tissue group was a few (+) immunopositive connective tissue cells and it ranged from no positive cells (0) to few to moderate (+/++) immunopositive cells (Figure 2D).

The Kruskal–Wallis H test indicated that a statistically significant difference was not present in the number of DLX4 immunopositive epitheliocytes among all four tissue groups (H=2.287, df=3, p=0.515). This test also revealed that a statistically significant difference was not present in the number of DLX4 positive cells in the connective tissues among all four tissue groups (H=1.839, df=3, p=0.606).

The Mann–Whitney U test indicated no statistically significant difference in the number of DLX4 immunopositive surface epitheliocytes between the controls and the unilateral cleft lip tissue group (U=122.5, p=0.906). This test revealed no statistically significant difference in the number of DLX4 immunopositive connective tissue cells between the controls and the unilateral cleft lip tissue group (U=122.5, p=0.899).

The Mann–Whitney U test indicated no statistically significant difference in the number of DLX4 immunopositive epitheliocytes between the controls and the bilateral cleft lip tissue group (U=40.0, p=0.862). This test indicated no statistically significant difference in the number of DLX4 immunopositive connective tissue cells between the controls and the bilateral cleft lip tissue group (U=42.5, p=0.796).

The Mann–Whitney U test revealed no statistically significant difference in the number of DLX4 immunopositive epitheliocytes between the controls and the cleft palate tissue group (U=68.5, p=0.449). This test indicated no statistically significant difference in the number of DLX4 immunopositive connective tissue cells between the controls and the cleft palate tissue group (U=79.5, p=0.585).

### FOXE1 immunohistochemistry

**Figure 3.**
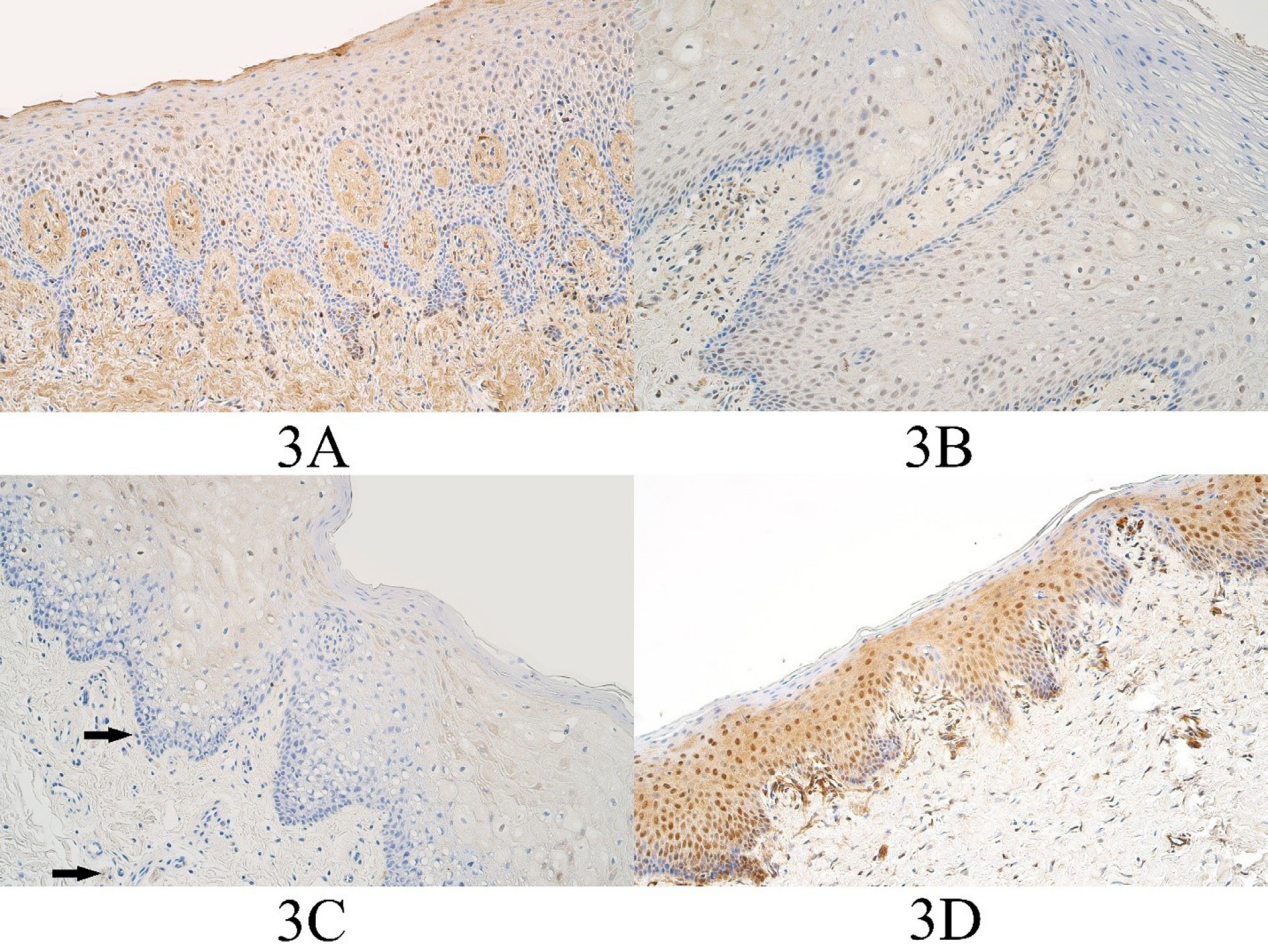
FOXE1 immunopositive cells within the control group and cleft affected tissues. (3A) Control with moderate to numerous FOXE1-containing epitheliocytes and few to moderate FOXE1-containing connective tissue cells, FOXE1 IMH, 200x. (3B) Unilateral cleft lip patient with a moderate number of FOXE1-containing epitheliocytes and the connective tissue cells, FOXE1 IMH, 200x. (3C) Bilateral cleft lip patient with a moderate number of FOXE1-containing epitheliocytes and a few FOXE1 positive connective tissue cells (arrows), FOXE1 IMH, 200x. (3D) Cleft palate patient with numerous FOXE1-containing epitheliocytes and few to moderate number of FOXE1 positive connective tissue cells, FOXE1 IMH, 200x.

In the control group the median number of FOXE1 positive epitheliocytes was few to moderate (+/++) and it ranged from barely detectable immunopositive epitheliocytes (0/+) to moderate (++) number of FOXE1 immunopositive cells. The median number of FOXE1 positive control group connective tissue cells was a few (+) and ranged from a rare occurrence (0/+) to few to moderate (+/++) number of FOXE1 immunopositive connective tissue cells (Figure 3A).

In the unilateral cleft lip tissue group the median number of FOXE1 positive epitheliocytes was moderate (++) and it ranged from a few (+) to numerous (+++) number of FOXE1 positive surface epitheliocytes. The median number of FOXE1 positive connective tissue cells in the unilateral cleft lip tissue group was moderate (++) and it ranged from a few (+) positive cells to numerous to abundant (+++/++++) FOXE1 positive cells (Figure 3B).

In the bilateral cleft lip tissue group the median number of FOXE1 positive cells in the surface epithelium was moderate to numerous – numerous (++/+++ - +++) and it ranged from a few (+) to numerous (+++) number of immunopositive epitheliocytes. In the bilateral cleft lip group connective tissue, the median number of FOXE1 structures was moderate to numerous (++/+++) and it ranged from no immunopositive structures (0) to numerous to abundant (+++/++++) immunopositive structures (Figure 3C).

In the cleft palate tissue group the median number of FOXE1 positive surface epitheliocytes was few to moderate (+/++) and it ranged from a few (+) to numerous (+++) immunopositive epitheliocytes. In the cleft palate group connective tissues, the median number of FOXE1 positive structures was a few to moderate (+/++) and it ranged from a few (+) to numerous (+++) immunopositive connective tissue cells (Figure 3D).

The Kruskal–Wallis H test revealed that there was no statistically significant difference among all four tissue groups in the number of FOXE1 immunopositive surface epitheliocytes (H=6.649, df=3, p=0.084). This test revealed that a statistically significant difference was present in the number of FOXE1 positive cells in the connective tissues among all four tissue groups (H=16,876, df=3, p=0.001).

The Mann–Whitney U test indicated no statistically significant difference in the number of FOXE1 immunopositive surface epitheliocytes between the controls and the unilateral cleft lip tissue group (U=52.0, p=0.118). This test revealed a statistically significant difference in the number of FOXE1 immunopositive connective tissue cells between the controls and the unilateral cleft lip tissue group (U=16.0, p=0.003).

The Mann–Whitney U test indicated no statistically significant difference in the number of FOXE1 immunopositive epitheliocytes between the controls and the bilateral cleft lip tissue group (U=26.5, p=0.696). This test indicated no statistically significant difference in the number of FOXE1 immunopositive connective tissue cells between the controls and the bilateral cleft lip tissue group (U=17.0, p=0.111).

The Mann–Whitney U test revealed no statistically significant difference in the number of FOXE1 immunopositive epitheliocytes between the controls and the cleft palate tissue group (U=52.0, p=0.631). This test indicated a statistically significant difference in the number of FOXE1 immunopositive connective tissue cells between the controls and the cleft palate tissue group (U=27.0, p=0.032).

### HOXB3 immunohistochemistry

**Figure 4.**
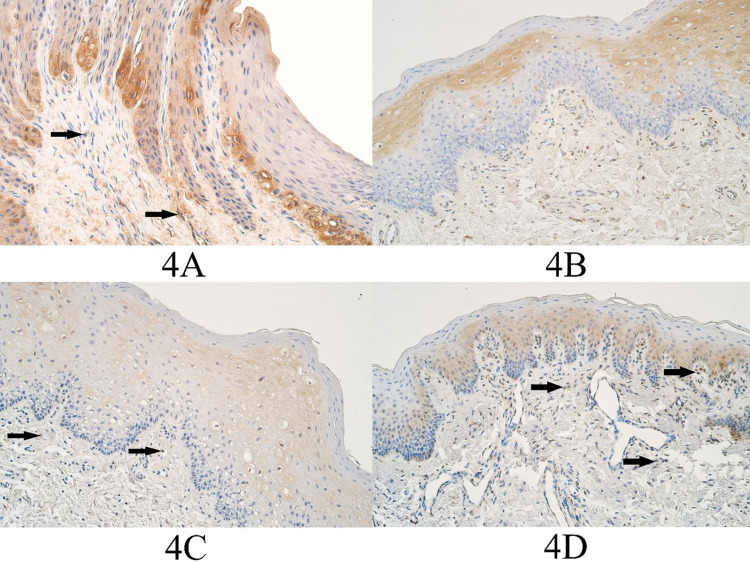
HOXB3 immunopositive cells within the control group and cleft affected tissues. (3A) Control group with moderate HOXB3-containing epitheliocytes and few positive connective tissue cells (arrows), HOXB3 IMH, 200x. (3B) Unilateral cleft lip patient with a moderate number of HOXB3-containing epitheliocytes and the connective tissue cells, HOXB3 IMH, 200x. (3C) Bilateral cleft lip patient with moderate to numerous weakly stained HOXB3 epitheliocytes and a few positive connective tissue cells (arrows), HOXB3 IMH, 200x. (3D) Cleft palate patient with a moderate number of HOXB3-containing epitheliocytes and few to moderate HOXB3 positive connective tissue cells (arrows), HOXB3 IMH, 200x.

In the control group the median number of HOXB3 positive epitheliocytes was a few (+) and it ranged from a few (+) to moderate (++) number of HOXB3 immunopositive cells. The median number of HOXB3 positive connective tissue cells in the control group was moderate (++) and ranged from few to moderate (+/++) to moderate (++) number of immunopositive connective tissue cells (Figure 4A).

In the unilateral cleft lip tissue group the median number of HOXB3 positive epitheliocytes was moderate to numerous (++/+++) and it ranged from a few (+) to numerous to abundant (+++/++++) HOXB3 positive surface epitheliocytes. The median number of HOXB3 positive connective tissue cells in the unilateral cleft lip tissue group was moderate (++) and it ranged from a few (+) positive cells to numerous to abundant (+++/++++) HOXB3 positive cells (Figure 4B).

In the bilateral cleft lip tissue group the median number of HOXB3 positive cells in the surface epithelium was moderate (++) and it ranged from a few (+) to moderate to numerous (++/+++) number of immunopositive epitheliocytes. In the bilateral cleft lip group connective tissues, the median number of HOXB3 structures was moderate (++) and it ranged from a few (+) to moderate to numerous (++/+++) immunopositive structures (Figure 4C).

In the cleft palate tissue group the median number of HOXB3 positive surface epitheliocytes was few to moderate to moderate (+/++ - ++) and it ranged from a few (+) to numerous (+++) immunopositive epitheliocytes. In the cleft palate group connective tissues, the median number of HOXB3 positive structures was few to moderate (+/++) and it ranged from a few (+) to numerous (+++) immunopositive connective tissue cells (Figure 4D).

The Kruskal–Wallis H test revealed a statistically significant difference among all four tissue groups in the number of HOXB3 immunopositive surface epitheliocytes (H=20.689, df=3, p<0.001). This test also revealed that a statistically significant difference was present in the number of HOXB3 positive cells in the connective tissue among all four tissue groups (H=8.419, df=3, p=0.038).

The Mann–Whitney U test indicated a statistically significant difference in the number of HOXB3 immunopositive surface epitheliocytes between the controls and the unilateral cleft lip tissue group (U=20.0, p=0.004). This test indicated no statistically significant difference in the number of HOXB3 immunopositive connective tissue cells between the controls and the unilateral cleft lip tissue group (U=56.5, p=0.160).

The Mann–Whitney U test indicated no statistically significant difference in the number of HOXB3 immunopositive epitheliocytes between the controls and the bilateral cleft lip tissue group (U=21.5, p=0.337). This test indicated no statistically significant difference in the number of HOXB3 immunopositive connective tissue cells between the controls and the bilateral cleft lip tissue group (U=26.5, p=0.527).

The Mann–Whitney U test revealed no statistically significant difference in the number of HOXB3 immunopositive epitheliocytes between the controls and the cleft palate tissue group (U=41.5, p=0.266). This test indicated no statistically significant difference in the number of HOXB3 immunopositive connective tissue cells between the controls and the cleft palate tissue group (U=60.0, p=0.783).

### MSX2 immunohistochemistry

**Figure 5.**
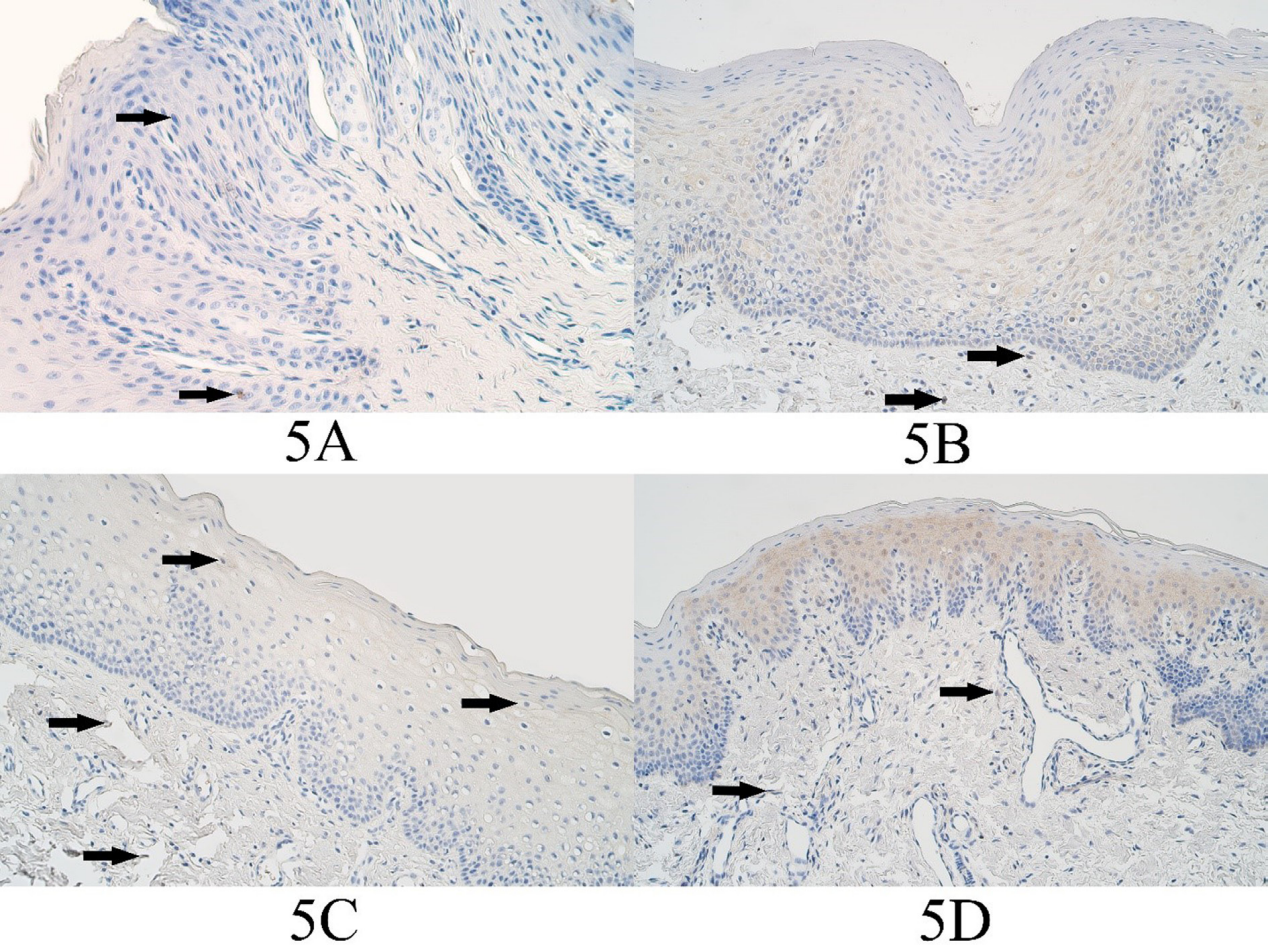
MSX2 immunopositive cells within the control group and cleft affected tissues. (3A) Control with a rare occurrence of MSX2-containing epitheliocytes (arrows) and absence of factor positive connective tissue cells, MSX2 IHC, 200x. (3B) Unilateral cleft lip patient with a moderate number of MSX2-containing epitheliocytes and a few positive cells in the connective tissue (arrows), MSX2 IHC, 200x. (3C) Bilateral cleft lip patient with a few MSX2-containing epitheliocytes (arrows) and a rare positive connective tissue cells (arrows), MSX2 IHC, 200x. (3D) Cleft palate patient with a moderate number of MSX2-containing epitheliocytes and with a few positive connective tissue cells (arrows), MSX2 IHC, 200x.

In the control group the median number of MSX2 positive epitheliocytes was no positive structures (0) and no MSX2 positive epitheliocytes could be found in any control group slides. In the connective tissues of the control group the median number of MSX2 positive structures was no positive structures (0) and it ranged from no positive structures (0) to a rare occurrence (0/+) of MSX2 positive cells (Figure 5A).

In the unilateral cleft lip tissue group the median number of MSX2 immunopositive epitheliocytes was moderate (++) and it ranged from no positive cells (0) to numerous (+++) MSX2 positive epithelial cells. In the unilateral cleft lip group connective tissues, the median number of MSX2 positive structures was a few (+) and it ranged from no positive structures (0) to moderate to numerous (++/+++) (Figure 5B).

In the bilateral cleft lip tissue group the median number of MSX2 positive cells in the surface epithelium was a few (+) and it varied from no immunopositive structures (0) to moderate to numerous (++/+++) immunopositive structures. The median number of MSX2 immunopositive structures in the bilateral cleft lip group connective tissues was a few (+) and it varied from no positive structures (0) to few to moderate (+/++) positive structures (Figure 5C).

In the cleft palate tissue group the median number of MSX2 positive surface epitheliocytes was a few (+) and it varied from no positive cells (0) to moderate (++) number of MSX2 positive epitheliocytes. The median number of MSX2 positive connective tissue cells in the cleft palate tissue group was a few (+) and it ranged from no positive cells (+) to few to moderate (+/++) MSX2 immunopositive connective tissue cells (Figure 5D).

The Kruskal–Wallis H test revealed a statistically significant difference among all four tissue groups in the number of MSX2 immunopositive surface epitheliocytes (H=30.652, df=3, p<0.001). This test also revealed that a statistically significant difference was present in the number of MSX2 positive structures in the connective tissues among all four tissue groups (H=10.233, df=3, p=0.017).

The Mann–Whitney U test indicated a statistically significant difference in the number of MSX2 immunopositive surface epitheliocytes between the controls and the unilateral cleft lip tissue group (U=7.5, p=0.001). This test indicated a statistically significant difference in the number of MSX2 immunopositive connective tissue cells between the controls and the unilateral cleft lip tissue group (U=21.0, p=0.004).

The Mann–Whitney U test revealed a statistically significant difference in the number of MSX2 immunopositive epitheliocytes between the controls and the bilateral cleft lip tissue group (U=10.0, p=0.021). This test indicated no statistically significant difference in the number of MSX2 immunopositive connective tissue cells between the controls and the bilateral cleft lip tissue group (U=14.0, p=0.056).

The Mann–Whitney U test revealed a statistically significant difference in the number of MSX2 immunopositive epitheliocytes between the controls and the cleft palate tissue group (U=27.5, p=0.045). This test indicated a statistically significant difference in the number of MSX2 immunopositive connective tissue cells between the controls and the cleft palate tissue group (U=24.5, p=0.023).

The semiquantitative evaluation of BARX1, DLX4, FOXE1, HOXB3, and MSX2 immunoreactivity is summarized in Table 2.

**Table 2.** Median values of semiquantitative evaluation for BARX1, DLX4, FOXE1, HOXB3, and MSX2 immunoreactivity within the control tissue group, unilateral cleft lip tissue group, bilateral cleft lip tissue group, and cleft palate tissue group.

	BARX1		DLX4		FOXE1		HOXB3		MSX2	
	E	CT	E	CT	E	CT	E	CT	E	CT
Controls	0	0	+	+	+/++	+	+	++	0	0
UCL	0	+	+/++	+	++	++	++/+++	++	++	+
BCL	0	+/++	+/++	+/++	++/+++ - +++	++/+++	++	++	+	+
CP	0	0/+ - +	+/++ - ++	+	+/++	+/++	+/++ - ++	+/++	+	+
H	0.000	8.511	2.287	1.839	6.649	16.876	20.689	8.419	30.652	10.233
p	1.000	0.037	0.515	0.606	0.084	0.001	<0.001	0.038	<0.001	0.017

Abbreviations: BARX1 – Homeobox Protein BarH-like 1; DLX4 – Distal-Less Homeobox 4; FOXE1 – Forkhead Box E1; HOXB3 – Homeobox Protein Hox-B3; MSX2 – Muscle Segment Homeobox 2; E – epithelium; CT – connective tissue; UCL – unilateral cleft lip, BCL – bilateral cleft lip, CP – cleft palate, H – Kruskal–Wallis H test statistic; p – p-value; 0 – no factor positive structures; 0/+ – a rare occurrence of factor positive structures; + – a few factor positive structures; +/++ – a few to moderate number of factor positive structures; ++ – a moderate number of factor positive structures; ++/+++ – a moderate to numerous factor positive structures; +++ – numerous factor positive structures, +++/++++ – numerous to abundant factor positive structures.

### Correlations

Spearman’s rank correlation coefficient calculation allowed to find multiple statistically significant correlations between the number of factor positive cells for BARX1, DLX4, FOXE1, HOXB3, MSX2 in the surface epithelium and the underlying connective tissues within each of the cleft affected tissue groups.

No statistically significant correlations between the evaluated factors were found in the control group.

### Correlations in the unilateral cleft lip tissue group

Multiple statistically significant positive correlations were calculated within the unilateral cleft lip tissue group.

Statistically significant strong correlations (r_s_=0.6–0.8) were notified between the number of HOXB3 immunopositive epitheliocytes and the number of HOXB3 immunopositive connective tissue cells (r_s_=0.682, p<0.001).

Statistically significant moderate correlations (r_s_ =0.4–0.6) were identified between the number of FOXE1 immunopositive connective tissue cells and the number of HOXB3 positive connective tissue cells (r_s_ =0.562, p<0.001), between the number of FOXE1 immunopositive epitheliocytes and the number of FOXE1 immunopositive connective tissue cells (r_s_ =0.544, p=0.001), between the number of DLX4 immunopositive epitheliocytes and the number of HOXB3 immunopositive epitheliocytes (r_s_ =0.460, p=0.005), between the number of FOXE1 immunopositive connective tissue cells and the number of HOXB3 positive epitheliocytes (r_s_ =0.455, p=0.005), between the number of MSX2 immunopositive epitheliocytes and the number of MSX2 immunopositive connective tissue structures (r_s_ =0.440, p=0.007), between the number of FOXE1 immunopositive epitheliocytes and the number of HOXB3 immunopositive connective tissue cells (r_s_ =0.432, p=0.009), between the number of HOXB3 containing connective tissue cells and the number of MSX2 containing connective tissue cells (r_s_ =0.422, p=0.010).

Statistically significant weak correlations (r_s_ =0.2–0.4) were notified between the number of HOXB3 containing epitheliocytes and the number of MSX2 containing epitheliocytes (r_s_ =0.392, p=0.018), between the number of BARX1 immunopositive connective tissue cells and the number of MSX2 containing epitheliocytes (r_s_ =0.391, p=0.018), between the number of DLX4 containing epitheliocytes and the number of FOXE1 containing connective tissue structures (r_s_ =0.378, p=0.023), between the number of FOXE1 containing epitheliocytes and the number of HOXB3 containing epitheliocytes (r_s_ =0.334, p=0.046).

The summary of statistically significant positive correlations within unilateral cleft lip tissue group can be found in Table 3.

### Correlations in the bilateral cleft lip tissue group

Multiple statistically significant positive and negative correlations were identified within the bilateral cleft lip tissue group.

Statistically significant strong positive correlations (r_s_ =0.6–0.8) were notified between the number of FOXE1 containing connective tissue cells and the number of HOXB3 containing epitheliocytes (r_s_ =0.798, p=0.002), between the number of HOXB3 containing epitheliocytes and the number of MSX2 containing epitheliocytes (r_s_ =0.785, p=0.003), between the number of FOXE1 immunopositive connective tissue structures and the number of MSX2 immunopositive epitheliocytes (r_s_ =0.773, p=0.003), between the number of DLX4 containing epitheliocytes and the number of DLX4 containing connective tissue cells (r_s_ =0.750, p=0.005).

**Table 3.** Statistically significant positive correlations in unilateral cleft lip tissue group (r_s_ - Spearman’s rho value).

Strength of correlation	Positive Correlations Between Factors in Unilateral Cleft Lip Affected Tissue	r_s_	p
Strong (0.6–0.8)	HOXB3 in epithelium and HOXB3 in connective tissue	0.682	<0.001
Moderate (0.4–0.6)	FOXE1 in connective tissue and HOXB3 in connective tissue	0.562	<0.001
	FOXE1 in epithelium and FOXE1 in connective tissue	0.544	0.001
	DLX4 in epithelium and HOXB3 in epithelium	0.460	0.005
	FOXE1 in connective tissue and HOXB3 in epithelium	0.455	0.005
	MSX2 in epithelium and MSX2 in connective tissue	0.440	0.007
	FOXE1 in epithelium and HOXB3 in connective tissue	0.432	0.009
	HOXB3 in connective tissue and MSX2 in connective tissue	0.422	0.010
Weak (0.2–0.4)	HOXB3 in epithelium and MSX2 in epithelium	0.392	0.018
	BARX1 in connective tissue and MSX2 in epithelium	0.391	0.018
	DLX4 in epithelium and FOXE1 in connective tissue	0.378	0.023
	FOXE1 in epithelium and HOXB3 in epithelium	0.334	0.046

**Table 4.** Statistically significant positive correlations in bilateral cleft lip tissue group (r_s_ - Spearman’s rho value).

Strength of correlation	Positive Correlations Between Factors in Bilateral Cleft Lip Affected Tissue	r_s_	p
Very strong (0.8–1.0)	FOXE1 in epithelium and MSX2 in epithelium	0.820	0.001
Strong (0.6–0.8)	FOXE1 in connective tissue and HOXB3 in epithelium	0.798	0.002
	HOXB3 in epithelium and MSX2 in epithelium	0.785	0.003
	FOXE1 in connective tissue and MSX2 in epithelium	0.773	0.003
	DLX4 in epithelium and DLX4 in connective tissue	0.750	0.005
Moderate (0.4–0.6)	FOXE1 in epithelium and FOXE1 in connective tissue	0.587	0.045
	DLX4 in epithelium and FOXE1 in connective tissue	0.582	0.047
	BARX1 in connective tissue and DLX4 in connective tissue	0.561	0.046

Statistically significant moderate correlations (r_s_ =0.4–0.6) were notified between the number of SHH immunopositive connective tissue cells and the number of WNT3A immunopositive epitheliocytes (r_s_ =0.591, p=0.043), between the number of FOXE1 containing epitheliocytes and the number of FOXE1 containing connective tissue cells (r_s_ =0.587, p=0.045), between the number of MSX2 immunopositive epitheliocytes and the number of SHH immunopositive epitheliocytes (r_s_ =0.586, p=0.045), between the number of DLX4 containing epitheliocytes and the number of FOXE1 immunopositive connective tissue structures (r_s_ =0.582, p=0.047), between the number of FOXE1 immunopositive connective tissue structures and the number of WNT3A immunopositive epitheliocytes (r_s_ =0.585, p=0.047), between the number of SHH immunopositive epitheliocytes and the number of SOX3 immunopositive epitheliocytes (r_s_ =0.580, p=0.048), between the number of BARX1 immunopositive connective tissue cells and the number of DLX4 immunopositive connective tissue cells (r_s_ =0.561, p=0.046).

The summary of statistically significant positive correlations within the bilateral cleft lip tissue group can be found in Table 4.

Statistically significant strong negative correlations (r_s_ = –0.8…–0.6) were identified between the number of BARX1 immunopositive connective tissue cells and the number of MSX2 immunopositive connective tissue cells (r_s_ =–0.783, p=0.002), between the number of BARX1 immunopositive connective tissue structures and the number of DLX4 immunopositive epitheliocytes (r_s_ =–0.615, p=0.033).

The summary of statistically significant negative correlations within bilateral cleft lip tissue group can be found in Table 5.

**Table 5.** Statistically significant negative correlations in biilateral cleft lip tissue group (r_s_ - Spearman’s rho value).

Strength of correlation	Negative Correlations Between Factors in Bilateral Cleft Lip Affected Tissue	r_s_	**p**
Strong (–0.8…–0.6)	BARX1 in connective tissue and MSX2 in connective tissue	–0.783	0.002
	BARX1 in connective tissue and DLX4 in epithelium	–0.615	0.033

### Correlations in the cleft palate tissue group

Multiple statistically significant positive correlations were identified within the cleft palate tissue group.

Statistically significant moderate correlations (r_s_ =0.4–0.6) were identified between the number of HOXB3 containing epitheliocytes and the number of HOXB3 positive connective tissue cells (r_s_ =0.557, p=0.005), between the number of DLX4 containing epitheliocytes and the number of FOXE1 containing epitheliocytes (r_s_ =0.534, p=0.007), between the number of FOXE1 positive epitheliocytes and the number of MSX2 positive epitheliocytes (r_s_ =0.432, p=0.040).

The summary of statistically significant positive correlations within the cleft palate tissue group can be found in Table 6.

**Table 6.** Statistically significant positive correlations in cleft palate tissue group (r_s_ - Spearman’s rho value).

Strength of correlation	Positive Correlations Between Factors in Cleft Palate Affected Tissue	r_s_	p
Moderate (0.4–0.6)	HOXB3 in epithelium and HOXB3 in connective tissue	0.557	0.005
	DLX4 in epithelium and FOXE1 in epithelium	0.534	0.007
	FOXE1 in epithelium and MSX2 in epithelium	0.432	0.040

## Discussion

### Immunohistochemical evaluation

Our study has noted multiple statistically significant differences in the numbers of factor positive cells between the cleft affected tissue and the controls.

While evaluating the number of BARX1 positive cells statistically significant differences were found between the control connective tissues and unilateral cleft lip connective tissues and between the control connective tissues and the bilateral cleft lip connective tissues. Interestingly, no statistically significant difference was found in the number of BARX1 positive connective tissue cells between the controls and the cleft palate tissue group which could indicate a locational (based on the cleft type) difference in the number of BARX1 positive connective cells within the postnatal mucosal connective tissue. BARX1 (the gene encoding BARX1 protein) expression has been previously described in orofacial mesenchymal tissue, mainly within the mesenchyme of the developing maxillary and mandibular regions and later in the dental papilla of developing molars [31, 32]. The presence of BARX1 in postnatal mucosal connective tissue which mainly develop from the orofacial ectomesenchyme could indicate a possible involvement and interaction of BARX1 with the formation of unilateral and bilateral cleft lip. No BARX1 positive cells were found in the surface epithelium of any control group or any cleft group patient slides which also coincides with previous research results in mice models where BARX1 gene expression mainly has been found in the underlying mesenchymal tissue and not in the developing oral cavity epithelium during teeth development [31].

For DLX4 no statistically significant differences were notified between the controls and any of the cleft affected tissue groups. While DLX4 did not show statistically significant differences in immunoreactivity between the tissue groups, its involvement with the formation of orofacial cleft could not be excluded because of its regulatory role during the development of the craniofacial region [12, 13].

The assessment of FOXE1 notified statistically significant differences in the number of FOXE1 containing connective tissue cells between the controls and the unilateral cleft lip tissue group and between the controls and cleft palate tissue group, but not between the control group and the bilateral cleft lip tissue group. Statistically significant differences for the number of FOXE1 containing cells were not found in the epithelium between the controls and any cleft tissue group epithelium. These results could indicate that FOXE1 might play a similar regulatory role within the developing connective tissue of the palatal region and the lip and the disruption of FOXE1 regulation could have a similar impact on the formation of cleft palate and unilateral cleft lip. FOXE1 gene expression previously has been described in the epithelium of the secondary palate which implies its regulatory purpose during palatogenesis process [32] and the loss of FOXE1 function has been associated with formation of cleft palate and cleft lip in humans [32, 33].

After evaluation of HOXB3 immunoreactivity the only statistically significant difference was notified in the number of HOXB3 containing surface epitheliocytes between the control group and the unilateral cleft tissue group. No other statistically significant differences for HOXB3 were notified. While HOXB3 gene involvement with the development of the craniofacial region has been previously described in mice [19], data about the HOXB3 protein presence in human tissue is relatively limited mainly discussing disruption of HOXB3 function in formation of some pathologies with altered cell proliferation like breast cancer [34] and glioblastoma [35]. The statistically significant difference of HOXB3 immunoreactivity between the control epithelium and unilateral cleft lip epithelium might indicate a possible pathogenetic involvement of this factor for this specific type of nonsyndromic facial cleft by possible alteration of the cell proliferation process within the unilateral cleft affected tissue.

Assessment of MSX2 immunoreactivity indicated statistically significant differences in the number of MSX2 positive cells in the epithelium between the controls and each of the cleft affected tissue groups while statistically significant differences were also found in the number of MSX2 containing connective tissue cells between the controls and the unilateral cleft lip tissue group and between the controls and the cleft palate tissue group but not between controls and bilateral cleft lip tissue group. This implies the possible role of MSX2 in formation of multiple orofacial cleft types. MSX2 is involved with the formation of orofacial structures like tooth development and affects the differentiation of the oral cavity epithelium into the tooth enamel organ [36, 37]. These previously described roles of MSX2 within the oral cavity epithelium could also imply a possible role of MSX2 during the formation of cleft affected tissue where the epithelial and mesenchymal tissue interactions are typically abnormal.

### Correlations

#### Unilateral cleft lip group

In the unilateral cleft lip patient group multiple statistically significant positive correlations were notified.

A statistically significant strong correlation was identified between the number of HOXB3-containing cells in the epithelium and connective tissue. HOXB3 gene involvement with craniofacial region morphogenesis has been previously described in mice [19] and together with other HOX genes it helps to regulate the migration of cranial neural crest cells [20]. The results from our study could indicate that HOXB3 might be involved with epithelial tissue and connective tissue interactions during the formation of the lip and disruption of HOXB3 function could be a part of unilateral cleft lip morphopathogenetic mechanisms.

Multiple statistically significant moderate correlations were identified in the unilateral cleft lip tissue group. The correlation between the number of FOXE1-containing epithelial cells and FOXE1-containing connective tissue cells could be an indication that FOXE1 might provide a regulatory role during the formation of the lip. FOXE1 has been previously described as a regulatory factor during craniofacial morphogenesis process and more information has been found about its regulatory role in other regions like in the development of the thyroid gland and thyroid gland pathologies with disrupted cell proliferation like thyroid cancer [38].

Other correlations have been found between FOXE1 and other factors. Some correlations between FOXE1 and HOXB3-containing cells could show a possible molecular interaction between these two factors in unilateral cleft lip tissue, for example, the correlation between the number of FOXE1-containing connective tissue cells and the number of HOXB3-containing connective tissue cells and the correlation between the number of FOXE1-containing epitheliocytes and HOXB3-containing connective tissue cells, between the number of FOXE1-containing connective tissue cells and HOXB3-containing epithelial cells. FOXE1 together with HOXB3 has been described as a transcription factor which plays a regulatory role in formation of craniofacial organs and tissues, for example, during the development of the thyroid gland where FOXE1 has a more direct role in regulation of thyroid gland growth and formation, while HOXB3 has a more indirect role during thyroid gland and craniofacial structure development process [39]. Disruption of both HOXB3 and FOXE1 regulatory role within the cleft affected tissue could also show that both factors might play a role during cell proliferation and tissue growth regulation processes within the epithelium and the underlying connective tissue of the developing lip. Interestingly, a statistically significant weak correlation between FOXE1-containing epitheliocytes and HOXB3-containing epitheliocytes was also notified. This weak correlation between FOXE1 and HOXB3-containing epitheliocytes could indicate that the main interactions between FOXE1 and HOXB3 might possibly happen within the underlying connective tissue where HOXB3 typically regulates the migration of cranial neural crest cells [20] which in turn could possibly affect the growth and differentiation of the cleft affected tissue in this region.

A statistically significant weak correlation involving FOXE1 was identified between the number of FOXE1-containing connective tissue cells and DLX4-containing epitheliocytes. The weak correlation between FOXE1 and DLX4 might be incidental but the regulatory role of both transcription factors is important for the craniofacial region in general. DLX genes help to determine the patterning of the developing mandible and maxillary region in mammals [15, 40] while disruption of FOXE1 has been associated with the formation of different orofacial cleft types, including cleft lip [40].

A statistically significant moderate correlation was found between the number of DLX4-containing epitheliocytes and HOXB3-containing epitheliocytes. DLX4 and HOXB3 are involved with the developing pharyngeal arch region but their exact role is relatively unclear due to simultaneous activity of other homeobox transcription factors within the developing orofacial region [41]. This interaction could indicate that DLX4 and HOXB3 could also provide a regulatory role within the developing lip.

A moderate correlation was found between MSX2-containing epitheliocytes and MSX2-containing connective tissue cells in unilateral cleft lip tissue. MSX2 involvement with the formation of orofacial clefts has been previously described [25] and MSX2 is involved with epithelial and connective tissue interactions during the development of the orofacial region [36, 37]. This could indicate that MSX2 might be involved with interactions between the developing oral cavity epithelium and the underlying connective tissue within the postanatal unilateral cleft lip tissue.

Some other statistically significant moderate and weak correlations involving MSX2 were identified – between the number of MSX2-containing connective tissue cells and HOXB3-containing connective tissue cells, between MSX2-containing epitheliocytes and HOXB3-containing epitheliocytes, between MSX2-containing epitheliocytes and BARX1-containing connective tissue cells. MSX2 has been previously described as transcription factor which can regulate cell proliferation in some pathological conditions like oral squamous cell carcinoma in which increased MSX2 activity has been associated with decreased cell proliferation and a tumor suppressor-like effect [42]. HOXB3 has also been associated with the regulation of cell proliferation and increased HOXB3 activity promoted cell proliferation in some pathological conditions like glioblastoma [36]. These effects of MSX2 and HOXB3 together with other transcription factors could also possibly affect cell growth and proliferation in postnatal cleft affected tissue of the unilateral cleft lip where the correct tissue growth and remodeling processes have been disturbed. BARX1 has also been described as a transcription factor involved in cell proliferation and epithelial–mesenchymal tissue interactions. Increased BARX1 activity has been associated with enhanced cell proliferation and migration activity within pathological tissue like clear cell renal cell carcinoma [43]. While the interactions of MSX2, HOXB3, and BARX1 in unilateral cleft lip tissue might still be unclear and these transcription factors might be affected by some other still unknown factors, their possible role could still be important for the understanding of pathogenic mechanisms of orofacial cleft formation.

#### Bilateral cleft lip group

Multiple statistically significant correlations were identified in the bilateral cleft lip patient group.

A statistically significant very strong correlation was found between FOXE1-containing epitheliocytes and MSX2-containing epitheliocytes. Both FOXE1 and MSX2 have been associated with the development of craniofacial malformations [44, 45]. FOXE1 and MSX2 could possibly indirectly interact with each other during orofacial development through a complicated interconnected network of signaling pathways which regulate craniofacial development – the wingless-type MMTV integration site family (WNT) signaling, bone morphogenetic protein (BMP) signaling and Sonic hedgehog (SHH) signaling pathway [4, 46, 47]. The fact that a very strong correlation has been found between FOXE1 and MSX2-containing epitheliocytes could indicate that these transcription factors might be involved with the formation of the bilateral cleft lip and the interactions within the epithelium might play a crucial role during cleft pathogenesis process for this type of cleft.

Multiple statistically significant strong correlations were identified in bilateral cleft lip tissue. Interestingly there were statistically significant strong correlations involving FOXE1 – between the number of FOXE1-containing connective tissue cells and the number of HOXB3-containing epitheliocytes, and between the number of FOXE1-containing connective tissue cells and MSX2-contatining epitheliocytes. A statistically significant correlation was seen also between the number of HOXB3-contating epitheliocytes and MSX2-contatining epitheliocytes. This could indicate a close interaction between three factors – FOXE1, HOXB3, and MSX2 within bilateral cleft lip tissue. All three transcription factors are involved with each other indirectly within a network of signaling pathways like WNT, BMP and SHH signaling during craniofacial development [46, 47]. These correlations could imply that the dysregulation of tissue growth and remodeling during bilateral cleft lip formation could be set by a complex chain of interactions between FOXE1, HOXB3, and MSX2.

A statistically significant strong correlation was identified between the number of DLX4-containing epitheliocytes and DLX4-containing connective tissue cells. DLX4 dysregulation has been associated with changes in cell proliferation in pathological tissue in the craniofacial region like nasopharyngeal carcinoma [48]. There could be a possibility that DLX4 dysfunction might affect tissue growth in postnatal bilateral cleft lip affected tissue by affecting the normal growth and remodeling processes in cleft affected epithelium and connective tissue.

Some statistically significant moderate correlations were notified. The correlation between the number of FOXE1-containing epitheliocytes and FOXE1-containing connective tissue cells in the bilateral cleft lip tissue was alike to the same correlation seen in unilateral cleft lip tissue. This could mean that the pathogenetic role of FOXE1 might be quite similar in both types of lip clefts. FOXE1 has been mainly described as a transcription factor which regulates cell differentiation and proliferation specifically in the thyroid gland [49] but FOXE1 involvement in different craniofacial malformations has also been described [17, 18, 40] where the role of FOXE1 as a regulator of cell proliferation and differentiation could be similar.

Another statistically significant moderate correlation was found between the number of DLX4-containing epithelial cells and FOXE1-containing connective tissue cells in bilateral cleft lip tissue. A similar statistically significant weak correlation was found between the number of DLX4-containing epithelial cells and FOXE1-containing connective tissue cells in unilateral cleft lip connective tissue which could indicate that both DLX4 and FOXE1 interactions could be similar in both types of cleft lip with a more significant interaction being present within bilateral cleft lip tissue. The possible action of DLX4 and FOXE1 on the development of the craniofacial region most likely could be the regulation of cell differentiation and proliferation [48, 49].

The statistically significant moderate correlation between the number of BARX1-containing connective tissue cells and the number of DLX4-containing connective tissue cells could indicate that the underlying connective tissue is the main possible location of BARX1–DLX4 interactions. Previous studies have shown that BARX1-containing cells and BARX1 activity has mainly been found in mesenchyme-derived tissue during oral cavity development [31, 32] and both BARX1 and DLX4 regulate jaw development [15, 32].

Interestingly, our study revealed that there were statistically significant negative correlations in the bilateral cleft lip tissue which were notified between BARX1 in the connective tissue and MSX2 in the connective tissue and between BARX1 in the connective tissue and DLX4 in the epithelium. BARX1 and MSX2 could possibly interact with each other during facial development. Both BARX1 and MSX2 are affected by the WNT signaling pathway during the development of the maxilla and this pathway could be inhibited by Dickkopf-related protein 1 (DKK1) which afterwards downregulates BARX1 and MSX2 genes [50]. Interestingly the presence of a statistically significant negative correlation between BARX1 and MSX2 positive structures in bilateral cleft lip affected connective tissue could possibly indicate a disruption of this specific regulatory mechanisms. The interaction between BARX1 and DLX4 has been previously described in epithelial–mesenchymal interactions in other pathologies with disrupted cell proliferation like clear cell renal cell carcinoma [43]. The exact mechanisms of BARX1 and DLX4 interaction within bilateral cleft lip affected tissue remain relatively unclear but characteristics and correlations between these factors could imply their possible role in bilateral cleft lip pathogenetic mechanisms.

#### Cleft palate group

Some statistically significant moderate correlations were identified in the cleft palate group.

A statistically significant moderate correlation was found between the number of HOXB3-containing epithelial cells and HOXB3-containing connective tissue cells in cleft palate tissue. A similar but stronger correlation was seen also in unilateral cleft lip tissue which could imply some similarities of HOXB3 regulatory role in the developing of both lip and palate. This correlation involving HOXB3 could mean that HOXB3 might affect tissue growth and cell migration in the craniofacial region which has been previously described [20] and these activities could possibly be a part of cleft morphopathogenetic mechanisms together with other regulatory factors during cleft development.

A statistically significant moderate correlation was found between the number of FOXE1-containing epithelial cells and DLX4-containing epithelial cells. This seems to imply that the epithelium could be an important location for molecular interactions between FOXE1 and DLX4 during cleft palate formation. Both FOXE1 and DLX4 regulate craniofacial region development [40] and interactions and dysregulation between these factors could affect cleft tissue formation in the developing of palate.

A statistically significant moderate correlation was found between the number of FOXE1-containing epithelial cells and MSX2-containing epithelial cells. Interestingly, a similar but a very strong correlation was seen in bilateral cleft lip tissue which might imply some similarities in possible pathogenetic mechanisms between the two cleft types. This could also indicate that FOXE1 and MSX2 could interact with each other within the oral cavity epithelium. FOXE1 and MSX2 could interact with each other through different signaling pathways like BMP and SHH signaling [46, 47].

#### Study limitations

One of the main limitations of this specific study is the application of only immunohistochemistry for the detection of BARX1, DLX4, FOXE1, HOXB3, and MSX2 proteins in control and cleft affected tissue groups. The application of additional methods like *in situ* hybridization and gene amplification could contribute more information for this specific study. Genetic studies could be a valuable addition to complement and elaborate our study results while possibly providing more definite conclusions on the involvement of evaluated factors in cleft formation. An important limitation for the effective use of genetic studies is the fact that it is difficult to find and follow up specific cleft patients over a long period of time because the tissue material has been gathered over a period of two decades since the start of cleft morphological research in our departments and additional agreements would be necessary for the implementation of genetic research. The application of other additional study techniques for the evaluation of cleft candidate genes and their coded proteins is planned in the foreseeable future. Another limitation which could impact the results is the size of the control tissue group which is rather small due to the difficult access to available tissue material because of ethical concerns regarding the collection of said tissue material. An additional possible limitation in our research has been the control group collected from older individuals than the cleft patient groups which complicates the assessment and the comparison between the evaluated groups. The tissue from children of patient groups was gathered before the age of primary dentition while controls had mixed dentition. Factors, including aging changes in tissue endotypes, changes in protein expression and location due to tissue growth in older children, could possibly affect the differences in tissue evaluation between the groups. Difficulties with obtaining control tissue from relatively healthy children with the same or similar age like the patient groups have limited and complicated our assessment of exact differences between the controls and patient groups. Practically it is very difficult to obtain relatively healthy oral cavity tissue not affected by clefts from a significant number of healthy children of such age due to the previously established ethical aspects including permissions from parents and the presence of significant surgical indications to obtain any tissue at all.

The evaluation of postnatal cleft affected tissue can describe the characteristics and specifics of craniofacial tissue growth and development after birth, but it cannot clearly describe morphopathogenetic changes in mechanisms which have occurred prenatally. The problematic aspects of obtaining prenatal cleft affected tissue is a limitation of this study and a possible comparison between prenatal and postnatal cleft affected tissue morphophatogenesis could be future research topic.

## Conclusions

1. The statistically significant increase of transcription factor HOXB3 within unilateral cleft lip affected tissue signifies their association with this specific type of cleft.
2. Transcription factors BARX1, FOXE1 are probably involved with the formation of both unilateral cleft lip and bilateral cleft lip morphopathogenesis processes within the postnatal cleft affected tissue.
3. The statistically significant increase of MSX2 containing cells in all evaluated cleft patterns might indicate its role in the formation of different cleft types.
4. Interactions between transcription factors BARX1, DLX4, FOXE1, HOXB3, and MSX2 within the postnatal cleft affected tissue types indicates the presence of similar pathogenetic signaling mechanisms within different cleft type variations with some variations which could be affected by other unknown factors regulating gene expression within the postnatal cleft affected tissue.
